# Aerobic Exercise with Mediterranean-DASH Intervention for Neurodegenerative Delay Diet Promotes Brain Cells' Longevity despite Sex Hormone Deficiency in Postmenopausal Women: A Randomized Controlled Trial

**DOI:** 10.1155/2022/4146742

**Published:** 2022-04-04

**Authors:** Marwa M. Elsayed, Ahmed Rabiee, Ghada E. El Refaye, Hany F. Elsisi

**Affiliations:** ^1^Department of Physical Therapy for Cardiovascular/Respiratory Disorders and Geriatrics, Faculty of Physical Therapy, Cairo University, Giza 11432, Egypt; ^2^Department of Internal Medicine, Faculty of Medicine, Cairo University, Giza 11562, Egypt; ^3^Department of Physical Therapy for Women's Health, Faculty of Physical Therapy, Cairo University, Giza 11432, Egypt

## Abstract

**Objective:**

To investigate the combined impact of aerobic exercise and Mediterranean-DASH Intervention for Neurodegenerative Delay (MIND) diet on brain cells longevity in spite of sex hormones deficiency in obese postmenopausal women.

**Design:**

A parallel randomized clinical trial. *Subjects/Patients*. Sixty-eight eligible postmenopausal women were randomly assigned to one of two groups, one experimental and one control. The participants' age ranged from 60 to 75 years, and their body mass index ranged from 30 to 39.9 kg/m^2^.

**Methods:**

An experimental group whose members followed moderate-intensity treadmill exercise three times/week for three months with MIND diet and a control group whose members followed the MIND diet only. In addition to serum sex hormones, pre- and post-12-week assessments were performed to measure serum sex hormones as well as cognitive and functional levels.

**Results:**

The experimental group showed after intervention highly significant changes (*p* < 0.01) in sex hormones, cognitive functions, and functional levels compared with the control group (*p* < 0.05). In addition, no correlation was found between the measured variables in both groups after intervention (*p* > 0.05).

**Conclusion:**

Aerobic exercise combined with the MIND diet improves cognitive and functional levels and substitutes sex hormones deficiency in postmenopausal women, which affects the longevity of brain health.

## 1. Introduction

Cognitive functions are brain skills that control the behavior of people. These functions are essential for daily life activities, especially in older adults who have a higher rate of cognitive disorders [1]. Cognitive problems range from mild cognitive impairment (MCI) to dementia, which reduces the quality of life (QoL) along with serious economic consequences [2]. Globally, around 47.5 million people suffer from dementia, in addition to an average of 7.7 million cases diagnosed every year. Women represent about 75% of the diagnosed cases [3]. In Egypt, females are commonly known to have cognitive disorders, especially after menopause [4], as the hormonal changes affect different cognitive functions like memory, language, visuospatial abilities, and verbal fluency [5]. Many studies have reported a positive correlation between the high risk of cognitive disorders and postmenopausal changes in sex hormones [6, 7].

Sex hormones are widely distributed in brain cells, including the cells of the cerebral cortex, amygdala, hypothalamus, and hippocampus, where they enhance brain activities in memory as well as emotional and rewarding circuits [8]. Furthermore, estrogen has remarkable effects on the brain where it induces the synthesis of neurotrophins and increases blood flow in addition to its antioxidant, anti-inflammatory, neurotrophic, and neuroprotective effects on brain tissues [9]. It indicated that a decrease in estrogen levels in postmenopausal women could cause a lipid metabolism disorder and increasing the risk of atherosclerosis. [10]. Therefore, exploring safe and noninvasive therapeutic interventions which may substitute the decrease of sex hormones after menopause is potentially required.

Regular exercise and a healthy diet are the main corners of a healthy lifestyle recommended to prevent cognitive decline and improve cognitive abilities in older adults [11]. Aerobic exercise has numerous biological and psychological effects, such as increasing the gray matter volume in the frontal and hippocampal regions; inducing the release of serotonin, norepinephrine, and neurotrophic factors; enhancing neuroplasticity with better synaptic neuron connections; enhancing the integrity and function of cerebrovascular vessels; and increasing blood flow with more oxygen and nutrition to brain centers through improving glucose and lipid metabolism, causing micromolecular changes to arouse cognitive improvement effects [1, 12].

The Mediterranean-DASH Intervention for Neurodegenerative Delay (MIND) diet, which is abundant in antioxidants and anti-inflammatory compounds like vitamin E, flavonoids, folate, and carotenoids, enhances cognitive performance by slowing brain aging caused by free radical damage and inflammatory cytokines [13]. Furthermore, thousands of nutrients and phytochemicals found in the mediterranean diet may help to reduce inflammatory/oxidative status, which is linked to telomere shortening and lower levels of sirtuin1 (an antiaging chemical participated in the body's reaction to oxidative stress and chronic inflammation) [14, 15]. In addition, it has favorable effects on blood pressure (because of the decreased sodium and saturated fats), blood glucose (because of higher consumption of fibers and lower sweets with calorie restriction), and lipid profile (because of the decreased intake of saturated fats). Moreover, diet restriction results in weight loss, which leads to better self-esteem and psychological status [16].

Postmenopausal women are at higher risk of cognitive decline, and this reflects negatively on functional performance and QoL. Therefore, the aim of the present study was to investigate the impact of aerobic exercise and the MIND diet on cognition and functional levels in relation to the change in sex hormones in older adult women after menopause. In this study, we hypothesized that aerobic exercise and the MIND diet are effective interventions to improve cognition and functional level in spite of sex hormones deficiency in postmenopausal women.

## 2. Methods

### 2.1. Design

The present study is a parallel randomized clinical trial according to consort guidelines, carried out from December 2020 to July 2021 after getting the approval of the Ethical Committee of the Faculty of Physical Therapy, Cairo University, Egypt (no. P.T.REC/012/002773). All the procedures in this study were performed according to the Helsinki Declaration. The included participants signed informed consents for participation after a full explanation of the study's purpose. The registration identifier number of this study is: NCT04492540 on https://register.ClinicalTrials.gov.

### 2.2. Participants

Eighty postmenopausal women were enrolled in the outpatient clinic of the Department of Obstetrics and Gynecology and Internal Medicine, Cairo University Hospitals, Egypt. After assessing and reviewing the medical examinations of these participants by the study physician (AR-coauthor), 68 women were found to be eligible to safely take part in exercise and MIND-low caloric diet. The inclusion criteria in this study are as follows: being in the postmenopausal stage (confirmed by estradiol concentration ≤ 32.2 pg/ml, total testosterone (TT) level between 0.12 and 0.60 ng/ml, free testosterone (FT) level ≤ 2.85 pg/ml, and absence of menstrual cycle for more than one year), suffering from MCI (confirmed by having a Rowland Universal Dementia Assessment Scale (RUDAS) assessment score ranging from 23 to 26 and duration of disease diagnosis ranging from 6 to 24 months), decreased functional activity level (confirmed by having a Functional Independence Measures Scale (FIM) score ranging from 90 to 111), age ranging from 60 to 75 years, body mass index (BMI) ranging from 30 to 39.9 kg/m^2^, and being a nonsmoker. All patients had sedentary lifestyles according to Godin's leisure time questionnaire (less than 14 units) [17]. The social demographic data of participants was as follows: the age ranged from 60 to 75 years old, the family income was moderate, and primary school had not been completed. All of the included women are married and have no occupation.

**Figure 1:**
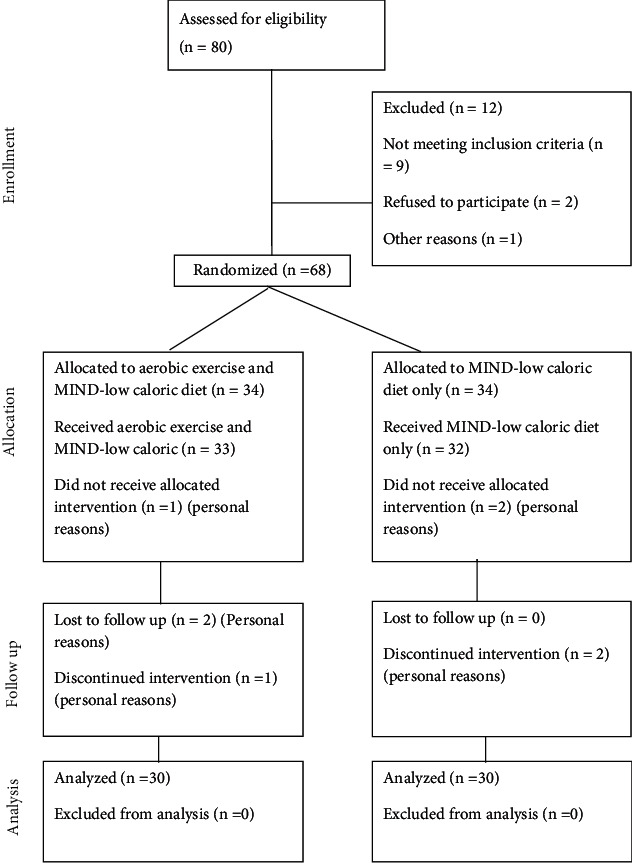
Consort flow diagram of the study.

Nine women were excluded according to the exclusion criteria: having diabetes mellitus, drinking alcohol, undergoing hormonal therapy, having any cardiovascular diseases, orthopedics or neurological disorders that affect exercise performance, participating in any weight reduction program at least six months before the study, and taking medications known to affect cognition, psychological status, and body weight. Two women refused to take part in the study, and one woman was excluded for other reasons. Finally, 60 women completed the intervention and all the assessments because eight women discontinued the study for personal reasons, as shown in Figure 1.

### 2.3. Intervention

#### 2.3.1. Exercise Protocol

The experimental group used a treadmill (Health-Mate HS M-Mt 195 V. Treadmill, China) to perform continuous moderate-intensity aerobic exercise three times per week for three months, following the recommendations of a recent international lipid expert panel on how to regularly train individuals who require statin therapy [18]. The training was done in the outpatient clinic of the Faculty of Physical Therapy, Cairo University, and supervised by the study physician and physiotherapists. The exercise session started with a 10-minute warm-up exercise in the form of light aerobic exercise. Then, each participant walked on the treadmill for 40 minutes until reaching the target intensity (60% of maximum heart rate (MHR) for the first 6 weeks, and then it was increased to 70% of MHR for the last six weeks (determined by submaximal graded treadmill exercise test according to the modified Bruce treadmill protocol [19] and assessed by using the RS400 Monitor). The final part of the session included a cooling down exercise similar to the warm-up one.

#### 2.3.2. Diet Program

The experimental and control groups followed an individualized MIND-low caloric diet according to the recommendations of the American Heart Association Nutrition Committee [20], which was prescribed by dieticians. Calories were restricted by reducing 20% of the participant's total daily energy expenditure (the total daily expenditure was determined for each participant individually according to the Harries Benedict equation) [21]. The MIND diet contained one serving of salad, other vegetables, one serving of nuts, and three servings of whole grains. Weekly, each participant was allowed to have two or more servings of berries (blueberries and strawberries referred), three-four servings of beans, one or more servings of fish, two servings of poultry at least, olive oil, and one serving or less of cheese, pastries, and sweets [22]. Also, participants were instructed to have only a tablespoon of butter per day, rarely eat red meat, and never eat margarine, as well as fried or fast food. The adherence of participants to a diet was evaluated by the Perceived Dietary Adherence Questionnaire. Individual sessions were carried out with a dietitian two times per week; written instructions were provided, and phone calls were available 24 hours a day.

### 2.4. Primary Outcomes

#### 2.4.1. Serum Sex Hormones

Blood samples were drawn before the beginning and after the completion of intervention (12 weeks) after at least 10 hours of fasting. Each participant's blood samples were analyzed in the same batch, and blood collection took place between 7 : 00 and 10 : 00 a.m. There is a chemiluminescence hormonal analysis for all hormones (estradiol, TT, FT, sex hormone binding globulin SHBG).

#### 2.4.2. Assessment of Cognitive Function

RUDAS is a scale evaluating 6 items: visuoconstructive praxis, verbal memory, motor praxis, visuospatial orientation, language, and judgment. The highest score is 30, which indicates the best cognition, while lower scores indicate lower cognitive impairment [23].

#### 2.4.3. Assessment Functional Activity Level

The FIM scale investigates the individual performance of daily life activities such as social cognition, self-care, locomotion, sphincter control, transfers, and communication. The score of independent performance starts with 1, which indicates total dependence, in contrast to 7, which indicates complete independence. Scores ranging from more than 1 to 5 reflect the need for assistance. The total score for the tasks performed was calculated; 126 represents the highest total score, and 18 represents the lowest independence level [24].

### 2.5. Secondary Outcomes

#### 2.5.1. Anthropometric Measures

Weight and height were determined through a physical examination using a Human Body Digital Height and Weight Scale AC110V-220 V 50HZ/60HZ. DHM-16/200/300, Zhengzhou to calculate BMI with this equation: BMI = body weight (kg)/height (m^2^).

### 2.6. Sample Size Calculation, Randomization, and Blinding

The sample size was detected before the study using G^∗^POWER statistical software (version 3.1.9.2; Franz Faul, Universitat Kiel, Germany) (two-tailed test: the difference between two independent means (two groups)). A sample size of 30 women per group (total = 60) was determined to allow the detection of an effect size (Cohen's *d*) of 0.53 with a 95% confidence interval. The significance level of 0.05 (type I error) for the outcome of cognitive functions was used.

For the randomization of the present study, random permuted blocks were used as 1 : 1 randomization. The size of blocks was 2, 4, and 6 to ensure that an equal number (*n* = 30) of participants per group was made through *R* Software (version 2.11). The sequence of random allocation was hidden in numbered envelopes showing the treatment assignment for each participant by a staff member not involved in the study. So, the participants and the study team were unaware of the assigned treatment.

### 2.7. Data Analysis

SPSS statistical software (version 25.0; IBM Corp., Armonk, New York, USA) was used for statistical analysis. The Shapiro–Wilk test was used to determine whether the data were distributed normally or not. The mean standard deviation (SD) was used to describe continuous data. Baseline characteristics between both groups were compared using the independent samples *t*-test. Dependent and independent sample *t*-tests were used to investigate the changes in variables pre and postintervention as well as the differences between the experimental and control groups, respectively.

Furthermore, Pearson's correlation coefficient and stepwise multiple linear regression were used to determine the correlation between the changes in cognitive and functional levels and sex hormones for each group, and *p* values less than 0.05 were considered statistically significant.

## 3. Results

The experimental and control groups showed no statistically significant differences in terms of weight, height, BMI, age, or serum concentration of sex hormones. The assessment of cognitive functions and functional activity at baseline (*p* > 0.05) is illustrated in Table 1.

In each of the experimental and control groups, there were statistically significant differences in BMI, weight, concentrations of sex hormones, cognitive functions, and FIM scores pre- and postintervention according to the paired sample *t*-test (*p* < 0.01, *p* < 0.05, respectively). Also, statistically significant differences were detected between the two groups in terms of BMI, weight, sex hormone concentrations, cognitive functions, and FIM scores postintervention according to the *p* independent *t*-test (*p* = 0.01) where the experimental group showed highly statistically significant differences compared with the control group as illustrated in Table 2.

Pearson's correlation was performed, followed by multiple linear regression analysis to determine whether the change in cognitive functions and functional levels was associated with the change in sex hormones.

In the experimental and control groups, no significant association was found between the mean difference in sex hormones, cognitive functions, and functional level assessments according to Pearson's correlation test (*p* > 0.05) (Table 3).

According to stepwise multiple linear regression, sex hormones had no significant effects on cognitive functions or functional levels after 12 weeks of training in the experimental group (*p* > 0.05) (Table 4).

No adverse events related to the intervention were reported in either group during the study.

## 4. Discussion

This study showed no correlation between the change in sex hormones and in cognitive and functional levels after a program of aerobic exercise with a MIND-low caloric diet or only the MIND-low caloric diet program. However, our results showed significant improvement in cognitive and functional levels despite the decreased levels of estrogen and testosterone after our approach. We believe that this is attributed to the beneficial effect of aerobic exercise and the MIND diet on improving cognition through different mechanisms than those previously introduced. This is similar to the effect of estrogen and testosterone on cognitive functions. This emphasizes that aerobic exercise and the MIND diet can successfully substitute for the deficiency of sex hormones in terms of improving cognition.

The results of this study showed a highly statistically significant reduction in weight, BMI, and serum levels of estradiol, TT, and FT as well as an increase in SHBG in the experimental group (*p* < 0.01) compared to the control group (*p* < 0.05). This finding agrees with the systematic review by Schmitz et al. [25], which showed that aerobic exercise and reduced caloric dietary increase SHBG levels and lower FT, TT, and estrogen levels [26]. As a result, preventing weight gain and lowering fat tissue is considered the major source of estrogen and androgens production after menopause. Weight loss leads to improving insulin sensitivity and declining its level which enhances the hepatic production of SHBG, leading to the increased reduction of estrogens and androgens levels [27].

Also, our results showed a highly statistically significant improvement in the RUDAS and FIM scales postintervention in the experimental group. This is consistent with the McAuley et al. [28] study, which demonstrated that a healthy diet and physical exercise reduced cognitive disorders due to improvements in neural systems and cells, resulting in better performance of general and specific executive functions even if the impairment of cognitive functions was caused by a variety of factors, including environmental and personal factors, both of which play important roles. Furthermore, Alghadi et al. [29] reported an improvement in the cognitive functions of older adults after applying different aerobic exercise intensities (moderate and high-intensity exercise), where the most beneficial modulation in memory and executive function takes place [30]. A previous systematic review by Brink et al. [31] showed that MIND, Mediterranean, and DASH diets improve cognition and decrease the risk of dementia. The most favorable outcomes resulted from the MIND diet.

In the introduction, we mentioned different mechanisms to explain the improvement in cognitive functions noticed in the elderly when performing aerobic exercise [1, 11, 12], which have protective effects against various brain diseases and enhance the recovery of brain cell injury [32]. This protective effect is due to the various changes in physiological aspects such as damage of brain cells, oxidative imbalance of cells, and the changes in hormones taking part in the process of apoptosis in different body cells [33], increasing the release of different factors essential for neural differentiation and memory function [34]. Additionally, exercise induces remarkable changes in the microstructure and microcirculation of brain cells, improves neural transmission [35], and decreases depressive symptoms as well as memory disorders [36].

Moreover, the MIND diet protects the brain tissues against oxidative stress and inflammation, inducing neurogenesis and neural signaling, which results in the increase of the volume of gray matter and inferior frontal gyrus, and consequently, neuronal plasticity in frontal-temporal brain regions [37]. Phytochemicals from fruits, vegetables, herbs, and spices have been shown to have relevant immunomodulatory and/or anti-inflammatory activities in the context of brain aging, potentially interfere with and regulate the normal function of cells, particularly neuronal components [38], and play a beneficial role in lipid metabolism, endothelial function, and oxidative stress pathways [39]. Furthermore, flavonoids have positive effects on the ratio of arachidonic acid to eicosapentaenoic acid, as well as physiological and antioxidant benefits [40].

### 4.1. Limitations

Despite our positive outcomes in this study, some limitations should be considered. The choice of participants was limited to women without any cardiovascular disorders or medical problems. Only illiterate and low-educated women participated for eligibility, but high-level educated women did not participate. Women with surgical menopause and those who are undergoing hormonal therapy were excluded, which may have limited variations in the study results. Also, only one specific diet program and one type of exercise with a specific intensity were applied. We recommend including a larger number of women with more variability in the demographic data to evaluate cognitive abilities and investigate different types and intensities of exercise as well as other diet programs. In addition, we recommend further studies on the effect of exercise and diet on other common diseases in women after menopause because of the hormonal change, for example, but not limited to, breast cancer and cardiovascular diseases.

## 5. Conclusion

Aerobic exercise combined with the MIND diet has a positive effect on cognition and functional levels and counteracts the change in sex hormones in the postmenopausal period. So, we reported these noninvasive therapeutic approaches in this study to gain better cognition and improve QoL for women after menopause.

## Figures and Tables

**Table 1:** Baseline characteristics of study participants.

Characteristics		Experimental group (*N* = 30)	Control group (*N* = 30)	*p* value
Age (yrs)	Mean ± SD	65.39 ± 2.83	65.13 ± 3.17	0.74
	Range	60.00-70.00	60.00-73.00	
Height (cm)	Mean ± SD	152.13 ± 4.07	152.17 ± 3.88	0.97
	Range	145.00-160.00	145.00-160.00	
Weight (kg)	Mean ± SD	83.01 ± 9.60	83.14 ± 9.00	0.87
	Range	67.30-99.60	67.50-98.30	
BMI (kg/m^2^)	Mean ± SD	35.77 ± 2.87	35.83 ± 2.74	0.94
	Range	30.00-39.50	30.10-39.70	
Sex hormones				
Estradiol (pg/ml)	Mean ± SD	27.69 ± 2.73	27.70 ± 2.88	0.99
	Range	23.00-33.90	22.90-33.80	
Total testosterone (ng/ml)	Mean ± SD	0.40 ± .09	0.40 ± .08	0.90
	Range	0.25-0.55	0.24-0.54	
Free testosterone (pg/ml)	Mean ± SD	1.93 ± .63	1.79 ± 0.68	0.42
	Range	0.65-2.84	.66-2.86	
SHBG (nmol/ml)	Mean ± SD	28.13 ± 5.45	27.81 ± 5.55	0.82
	Range	20.40-39.10	20.20-39.20	
Cognitive functions and functional level				
RUDAS	Mean ± SD	24.07 ± 1.08	24.23 ± 1.10	0.55
	Range	23.00-26.00	23.00-26.00	
FIM	Mean ± SD	99.40 ± 6.55	100.20 ± 6.56	0.63
	Range	90.00-111.00	90.00-111.00	

BMI: body mass index; FIM: functional independence measure; RUDAS: Rowland Universal Dementia Assessment Scale; SHBG: sex hormone binding globulin. Data are represented as mean ± standard deviation (SD), which are statistically significant at *p* ≤ 0.05.

**Table 2:** Comparison of weight, BMI, sex hormones and cognitive functions, and functional level before and after 12 weeks, in both groups.

Variables	Experimental group (*N* = 30)		*p* paired *t*-test value	Control group (*N* = 30)		*p* paired *t*-test value	*p* indep.*t*-test value
	Pre	Post		Pre	Post		
Weight (kg) BMI (kg/m^2^)	83.01 ± 9.60 35.77 ± 2.87	73.62 ± 9.21 31.67 ± 2.85	< 0.01^∗^ < 0.01^∗^	83.14 ± 9.00 35.83 ± 2.74	79.78 ± 9.00 33.49 ± 2.80	< 0.05^∗^ < 0.05^∗^	0.01^∗^ 0.01^∗^
Sex hormones							
Estradiol (pg/ml) Total testosterone (ng/ml)	27.69 ± 2.73 0.40 ± 0.09	22.29 ± 2.96 0.30 ± 0.08	< 0.01^∗^ < 0.01^∗^	27.70 ± 2.88 0.40 ± 0.08	25.23 ± 3.00 0.37 ± 0.08	< 0.05^∗^ < 0.05^∗^	0.01^∗^ 0.01^∗^
Free testosterone (pg/ml) SHBG (nmol/ml)	1.93 ± 0.63 28.13 ± 5.45	1.34 ± 0.50 34.54 ± 6.71	< 0.01^∗^ < 0.01^∗^	1.79 ± 0.68 27.81 ± 5.55	1.65 ± 0.62 30.25 ± 5.88	< 0.05^∗^ < 0.05^∗^	0.01^∗^ 0.01^∗^
Cognitive functions and functional level							
RUDAS FIM	24.07 ± 1.08 99.40 ± 6.55	26.57 ± 1.17 112.53 ± 5.92	< 0.01^∗^ < 0.01^∗^	24.23 ± 1.10 100.20 ± 6.56	25.07 ± 0.94 106.07 ± 6.43	< 0.05^∗^ < 0.05^∗^	0.01^∗^ 0.01^∗^

BMI: body mass index; FIM: functional independence measure; RUDAS: Rowland Universal Dementia Assessment Scale; SHBG: sex hormone binding globulin. Data are represented as mean ± standard deviation (SD) Statistically significant at *p* ≤ 0.05.

**Table 3:** The association among changes (post-pre) in sex hormones and cognitive functions and functional level, in both groups.

Variables	Experimental group				Control group			
	RUDAS		FIM		RUDAS		FIM	
	*r* value	*p* value	*r* value	*p* value	*r* value	*p* value	*r* value	*r* value
Estradiol (pg/ml)	-0.16	0.37	-0.01	0.92	-0.03	0.85	-0.22	0.24
Total testosterone (ng/ml)	-0.19	0.29	-0.12	0.51	-0.08	0.66	-0.10	0.57
Free testosterone (pg/ml)	-0.33	0.07	-0.17	0.35	-0.26	0.16	-0.14	0.45
SHBG (nmol/ml)	0.04	0.81	0.03	0.84	0.01	0.98	0.02	0.89

Correlation is significant at *p* ≤ 0.05 according to Pearson's correlation coefficient. FIM: functional independence measure; RUDAS: Rowland Universal Dementia Assessment Scale; SHBG: sex hormone binding globulin.

**Table 4:** Multiple linear regression model showing the association among changes (post-pre) in sex hormones and cognitive functions and functional level, in both groups.

Variables	Experimental group				Control group			
	RUDAS		FIM		RUDAS		FIM	
	*B*	*p* value	*B*	*p* value	*B*	*p* value	*B*	*p* value
Estradiol (pg/ml)	0.139	0.479	0.492	0.363	.121	0.449	0.699	0.041^∗^
Total testosterone (ng/ml)	-10.056	0.253	-10.712	0.654	-7.095	0.603	18.995	0.501
Free testosterone (pg/ml)	1.085	0.205	-2.802	0.232	1.580	0.108	2.882	0.153
SHBG (nmol/ml)	0.046	0.734	0.168	0.650	0.173	0.458	0.868	0.079

Values are presented as beta coefficient and *p* value of each parameter. ^∗^*p* value <0.05 is considered statistically significant according to multiple regression analysis. FIM: functional independence measure; RUDAS: Rowland Universal Dementia Assessment Scale; SHBG: sex hormone binding globulin.

## Data Availability

The data that support the study's findings are available from the corresponding author upon request.
